# Is There ‘Anther-Anther Interference’ within a Flower? Evidences from One-by-One Stamen Movement in an Insect-Pollinated Plant

**DOI:** 10.1371/journal.pone.0086581

**Published:** 2014-01-27

**Authors:** Ming-Xun Ren, Zhao-Jun Bu

**Affiliations:** 1 Key Laboratory of Aquatic Botany and Watershed Ecology, Wuhan Botanical Garden, Chinese Academy of Sciences, Wuhan, China; 2 Institute for Peat and Mire Research, Northeast Normal University, State Environmental Protection Key Laboratory of Wetland Ecology and Vegetation Restoration, Changchun, China; Centro de Investigación y de Estudios Avanzados, Mexico

## Abstract

The selective pressure imposed by maximizing male fitness (pollen dispersal) in shaping floral structures is increasingly recognized and emphasized in current plant sciences. To maximize male fitness, many flowers bear a group of stamens with temporally separated anther dehiscence that prolongs presentation of pollen grains. Such an advantage, however, may come with a cost resulting from interference of pollen removal by the dehisced anthers. This interference between dehisced and dehiscing anthers has received little attention and few experimental tests to date. Here, using one-by-one stamen movement in the generalist-pollinated *Parnassia palustris*, we test this hypothesis by manipulation experiments in two years. Under natural conditions, the five fertile stamens in *P. palustris* flowers elongate their filaments individually, and anthers dehisce successively one-by-one. More importantly, the anther-dehisced stamen bends out of the floral center by filament deflexion before the next stamen's anther dehiscence. Experimental manipulations show that flowers with dehisced anther remaining at the floral center experience shorter (1/3–1/2 less) visit durations by pollen-collecting insects (mainly hoverflies and wasps) because these ‘hungry’ insects are discouraged by the scant and non-fresh pollen in the dehisced anther. Furthermore, the dehisced anther blocks the dehiscing anther's access to floral visitors, resulting in a nearly one third decrease in their contact frequency. As a result, pollen removal of the dehiscing anther decreases dramatically. These results provide the first direct experimental evidence that anther-anther interference is possible in a flower, and that the selection to reduce such interferences can be a strong force in floral evolution. We also propose that some other floral traits, usually interpreted as pollen dispensing mechanisms, may function, at least partially, as mechanisms to promote pollen dispersal by reducing interferences between dehisced and dehiscing anthers.

## Introduction

In the current resurgence of pollination biology, the role of male reproductive successes (pollen transfer to the conspecific stigmas) in floral adaptation and evolution has been frequently emphasized in both theoretical [1], [2], [3] and experimental studies [4], [5], [6]. To enhance male reproductive success, one flower always has several male organs (stamens) with large quantities of male gametes (pollen). Despite having several stamens, pollen loss during the pollination process is very high, with less than 1% of the pollen removed reaching conspecific stigmas [6], [7]. Because of this significant waste, many flowers have evolved to present their pollen separately rather than all at once, to maximize the amount of pollen donated to stigmas [7], [8], [9]. Staggered dehiscence of anthers in the same flower is one of the most widespread mechanisms to present pollen sequentially to pollinators, which is known as one type of pollen ‘packaging’ and ‘dispensing’ mechanisms [8], [9], [10]. However, the advantage of such separated dehiscence of anthers may be accompanied with a cost. When the previous anther has finished its pollen presentation, it could block the contact of the next dehiscing anthers from pollinators since the optimal spatial position for pollen precise placement on pollinator's body is always restricted [8], [10].

Such interference between dehisced and dehiscing anthers has received little attention in current pollination biology, although the ‘anther-stigma interference’ (also known as ‘pollen-stigma interference’ [11], [12] or ‘male-female interference’ [13], [14] has been widely acknowledged and experimentally supported. In hermaphroditic flowers, both male and female organs may block each other's access to pollinators, thus leading to anther-stigma interference [14]. For example, the pistil may physically prevent effective pollen pick-up by pollinators from the stamens and, on the other hand, the numerous stamens may impede proper pollen deposition on stigmas by affecting pollinator positioning or shedding self pollen on stigmas [13], [14]. Similarly, it is possible that there is a conflict among anthers because in a flower, usually many stamens are presented simultaneously.

In this study, we used floral manipulations to test the role of stamen movement in reducing pollination interference by the dehisced anthers, using *Parnassia palustris* (Celastraceae [15]) as a model. We examined two main predictions of this hypothesis: (i) the one-by-one filament elongation and anther dehiscence is a special type of ‘pollen dispensing mechanism’ that prolongs the male phase of the flower; (ii) preventing the dehisced anther from bending away from the floral center will negatively affect pollen removal from the dehiscing anther.

## Materials and Methods

### Ethics statement

The locations for our field studies were protected areas. All necessary permits for the field studies were obtained from the Administration Bureau of Hani Nature Reserve issued to M.X. Ren, Z.J. Bu and W. Li. The studied species is not an endangered or protected species. Our field observations and experiments did not collect any plant, insect, or animal specimen.

### Study system

**Figure 1:**
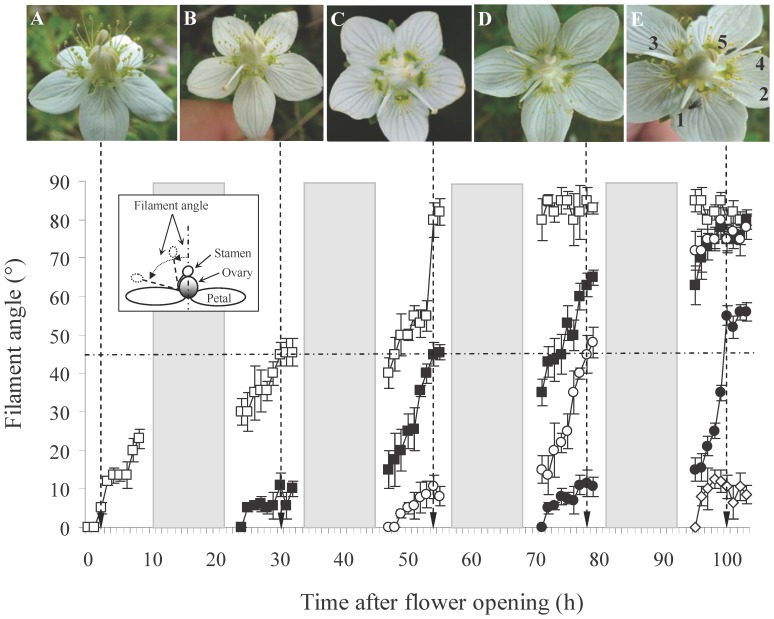
One-by-one stamen movement in *Parnassia palustris*. The first stamen (□) elongates its filament slowly and its anther starts dehiscence after about one day later when the filament reached its maximum length (A). The filament deflexes about one day later and the stamen bends out of the floral center (filament angle ≧45°, indicated by the horizontal dash line); simultaneously the second stamen (▪) begins its filament elongation (B). Similarly, the third (○), fourth (•), and fifth (◊) stamens start their filament elongation only after the former stamen has moved out of the floral center (C, D, E). The number next to each stamen in (E) indicates the movement sequence (note that the dehisced anthers always abscise during the movement process). Shaded areas indicate night-time.

*Parnassia palustris* is distinct for its single slender stem with single flower on the top and five strikingly-branched staminodes with a nectary-like tip on each branch [22], [23] (Fig. 1). The nectary-like tip has been proven to be attractant to pollinators [22]. There are five fertile stamens aggregating at the flower's center when the flower opens. These stamens become elevated, one-by-one through filament elongation, and then the anthers dehisce sequentially over the immature pistil [16]. Of greater significance is that the anther-dehisced stamen will bend out of the floral center via filament deflexion before the next stamen's filament elongation and anther dehiscence [17]. This appears to be one of the most complex types of stamen movement in angiosperms [17], [18], which can be named as ‘successive’ or ‘one-by-one’ stamen movement [17]. It is found not only in *P. palustris* and other species of the genus [17], but also in three other geographically and phylogenetically distant families: Rutaceae [18], Loasaceae [5], [19], [20], [21], and Tropaeolaceae (see review by Ren [17]), suggesting possible adaptation(s) underlying its evolution.

*P. palustris* is a widespread herb with a circumpolar distribution in Europe, Asia, and North America [22], [23]. It occurs in various open and moist habitats including peatlands, streamsides, and shaded places in valleys [22], [23]. This study was conducted in a large peatland (42°13′ N, 126°31′ E, elevation 900 m a.s.l.) in Hani National Nature Reserve on the southwestern side of Changbai Mountain, northeast China. The peatland covers about 1678 ha and with an approximate density of *P. palustris* of 10 individuals per m^2^. The coexisting dominant species are *Sphagnum* spp. (Sphagnaceae), *Carex lasiocarpa* (Cyperaceae), *Betula fruticosa* var. *ruprechtiana* (Corylaceae), *Potentilla fruticosa* (Rosaceae), and *Larix olgensis* (Pinaceae).

### Pattern of stamen movement

To determine the movement patterns of the stamens, we consecutively observed 35 flowers in both 2009 and 2012 respectively. The movement patterns were determined mainly by in situ observation with the help of a digital camera (Canon EOS 550D). Five main processes that characterize the stamen movements were recorded and calculated: (1) time for the first stamen to initiate filament elongation after the flower has become fully opened; (2) time for a stamen to initiate anther dehiscence after its filament elongation; (3) time for a stamen to initiate filament deflexion after the start of anther dehiscence (time for anther dehiscing at the floral center); (4) time for a stamen to finish its filament deflexion; (5) time for a stamen to initiate its filament elongation after the start of the former stamen's anther dehiscence. To further examine the movement pattern of the stamen in 2012, we used a protractor to measure the separation angle between the moving filament and the vertical direction at the floral center (filament angle) every hour after the flower had become fully opened. When the filament angle was ≧45°, its anther could hardly be touched by floral visitors and we determined this stamen as ‘moved out of the floral center’.

### Pollen production and breeding systems

To assess the number of pollen grains produced by each anther, we collected all five fertile anthers from 20 mature flowers (flowers opened but anthers not dehisced). Each anther was collected after the elongation of its filament, which indicates the anther is mature. The collected anthers were dissected separately and washed with 1 mL of distilled water to dislodge pollen grains from the anther. The 1-mL suspension was stirred in a vortex mixer for 30 s and then the number of pollen grains was counted in ten 1 µL samples of suspension, under a dissecting microscope (×200; Olympus SZX7). The number of pollen grains per anther was then calculated (*P*). The number of ovules were also counted under the microscope.

To determine the breeding system of *P. palustris*, we carried out four pollination treatments on 80 randomly selected flowers: (1) open pollination: 20 flowers were left for natural pollination without any treatment as a natural control. (2) bagged: 20 flowers were bagged before they were fully opened; (3) selfed: 20 flowers were hand-pollinated with self pollen from the last dehiscing anthers in the same flower; (4) crossed: 20 flowers were hand-pollinated with pollen from another individual. Flowers subjected to hand pollination were bagged with nylon mesh before anthesis and bagged again after hand pollination.

### Manipulation experiments

In 2009 and 2012, 50 and 80 flowers (plants) respectively were chosen at random in the middle of the population. To ensure that the flowers were not from the same individual and to avoid possible effects of density, the distance between every two experimental flowers was no less than 0.5 m. To ensure that the data were comparable among flowers and between years, we focused on the pollen removals of the second elongating stamen.

Among the selected flowers, half (25 and 40 in 2009 and 2012, respectively) were used as natural controls. Stamens in these flowers were allowed to move freely and be visited naturally by insects. The insect visit rate (number of insect visits per hour) and visitation duration (time for a single visit) were recorded. When the second-moved stamen deflexed its filament and moved the anther out of the floral center (filament angle ≧45°) we took off the anther and counted the remaining pollen grains (*P_1_*) under a dissecting microscope. Pollen removal under natural condition was calculated as *P* – *P_1_*.

For the other flowers, when the first stamen began to deflex after its free filament elongation and anther dehiscence, we stopped the deflexion by fixing the filament at the top (below the anther) with a white thread. The thread was tethered to the pedicel through the slit between the two opposite petals (Fig. S2). Thread and petal colour were matched to minimize the possible impact of the thread on insect visitation. The dehisced anther thus remained at the floral center. The next stamen (second stamen) was allowed to move freely and be visited naturally by insects. Insect visitation rate and duration were then recorded. When the second stamen moved out of the floral center we removed the anther and counted its remaining pollen grains (*P_2_*) under a dissecting microscope. Pollen removal under experimental manipulation was then calculated as *P* – *P_2_*. We also tethered a white thread to each control flower at the pedicel (Fig. S2) to both avoid the possible effect of floral manipulation and to help us locate these plants at the end of experiments.

### Insect visitations and behaviour

Insect visitations and behavior in natural and experimental flowers were surveyed in both years. The floral visitors were classified into different functional groups rather than individual species because functional group is easier and more accurate to record, and also because within each functional group members normally interacted with floral parts in a similar way [18], [24], [25]. Five functional groups were identified as floral visitors: hoverflies, ichneumon wasps, vespid wasps, flies, and ants. The main species of each functional group are shown in Fig. S1.

In the experimental periods, we observed floral visitors at local time of 0900–1000, 1200–1300, and 1400–1500 h for 15 labeled flowers among the natural and experimental flowers. The number of visits per hour and visitation duration (s) for a single visit were recorded for each functional group. We observed the behaviors of floral visitors with the help of a digital camera. Special attention was paid to the insect's contact frequency with the dehisced and dehiscing anthers.

### Statistical analyses

All the analyses were carried out using SPSS v.17.0 for Windows (SPSS Inc., USA). For data showing a normal distribution, such as pollen and ovule numbers and percentage of pollen removed, we used Student's *t*-test to test for possible differences between the two years and between manipulated and control treatments. We also used Student's *t*-test to compare each period of stamen movement between the two years. For data with random distribution, such as insect visitation rate, duration and contact frequency with the dehiscing anther, we firstly used Multivariate Analysis of Variation (MANOVA) to demonstrate the overall effect of insect visitation rate and duration. Then we used one-way MANOVA to test for differences in insect visitation rate and duration between manipulated and control treatments for each pollinator functional group in each year. To control for type-I error rate, we performed a sequential bonferroni adjustment of *P*-value to be 0.025 when we carried out the comparisons for pollinator visitation rate and duration.

## Results

### Floral biology and pattern of one-by-one stamen movement

Anthers of *P. palustris* flowers are pollen-rich, with 35640±4292 (mean ± SE) and 62635±1484 pollen grains per anther in 2009 and 2012, respectively. The number of ovules in a flower showed less variation between the two years, with 257±10 and 335±41 in 2009 and 2012, respectively. These results are reported in Table S1.

At the beginning of anthesis, all five fertile stamens were incurved and aggregated around the gynoecium. About 5 h after the flower had fully opened (286±13 min in 2009 and 313±12 min in 2012; N = 35 in each years), one stamen began to elongate its filament and to uplift its anther slowly above the gynoecium (Fig. 1A). It took approximately 24 h (1400 min) for the stamen to begin anther dehiscence after the start of filament elongation (Table 1). The anther dehiscence lasted for about 5 h (Table 1) after which the filament began to deflex and the filament angle increased as a result (Fig. 1B). It took about 24 h for the filament to finish its deflexion (Table 1), i.e. when the filament angle reached its maximum (Fig. 1C). The next stamen's filament elongation normally started only after the former stamen had moved out of the floral center (filament angle ≧45°) (Fig. 1), which was more than 3.5 hours after the start of the former stamen's anther dehiscence (Table 1). The five fertile stamens moved in an alternate sequence (as opposed to a clockwise or an anti-clockwise sequence; Fig. 1E). For every movement stage there was no significant difference between the two years (*P*>0.055, Student's *t*-test; Table 1).

**Table 1:** Patterns of one-by-one stamen movement (min) in *Parnassia palustris* flowers.

Year	Time 1	Time 2	Time 3	Time 4	Time 5
2009 (N = 35)	286±12.5	1476±51	295±16	1596±95	248±9
2012 (N = 35)	313±12	1361±55	317±15	1408±45	220±11
*t*-test	0.130	0.128	0.317	0.083	0.055

None of the movement stages between the two years were different significantly at p<0.05 (Student's *t*-test).

Data are Mean ± S.E. Time 1, time for the first stamen to start filament elongation after the flower has becomes full opened. Time 2, time for the filament to reach its maximum length, i.e. start of anther dehiscence. Time 3, time for a stamen to begin its filament deflexion after the start of anther dehiscence (time for anther dehiscence). Time 4, time for a stamen to finish its filament deflexion. Time 5, time for the next stamen to begin its filament elongation after the start of the former stamen's anther dehiscence.

**Figure 2:**
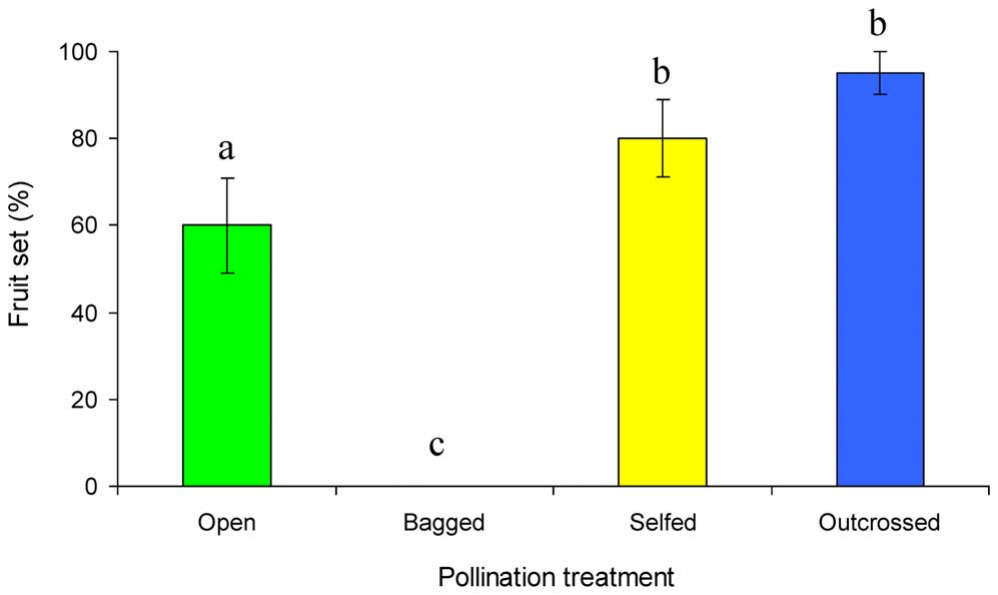
Breeding system of *Parnassia palustris*. Different letters above the bars indicate statistically significant difference at P<0.05 (one-way ANOVA, N = 20). Open, open-pollination; Bagged, bagged without any treatment; Selfed, hand self-pollination (hand-pollinated by the self pollen from the last-dehisced anther); Outcrossed, hand cross-pollination.

The results of our tests on the breeding system of *P. palustris* are shown in Fig. 2. Under natural conditions, fruit set was 60%±10.95% (N = 20). There was no significant difference in fruit set between hand self-pollination (80%±8.94%. N = 20) and cross-pollination (95%±4.87% N = 20), indicating that *P. palustris* is highly self-compatible and there is pollen limitation under natural conditions (Fig. 2). The rate of fruit set in bagged flowers without any treatment was zero (N = 20), which indicates that there is no spontaneous autogamy in this species.

### Pollinator behaviours and pollination consequences

**Figure 3:**
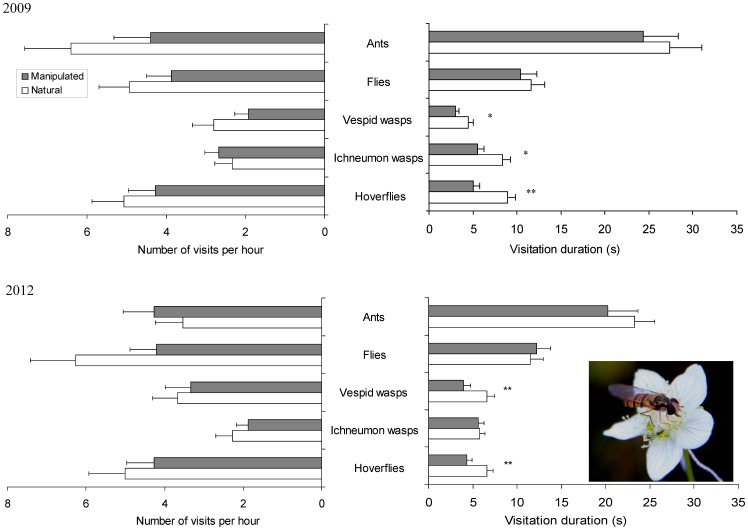
Visitation rate and duration of floral visitors to natural and manipulated flowers in two years. Data are Mean ± SE (“*” *P*<0.025; “**” *P*<0.005; one-way ANOVA). The inserted picture shows the most common pollinator, a hoverfly, visiting a manipulated flower.

The five insect functional groups on *P. palustris* flowers showed different forage preferences: hoverflies seek both nectar and pollen, vespid wasps only seek pollen, ichneumon wasps and ants feed only on nectar at the base of filament and flies mainly forage the nectar on the top of staminodes (Fig. S1). These insects differed in visitation rate and duration (Table 2), with hoverflies, flies and ants as the most common visitors to the flowers (Fig. 3)

**Table 2:** Multivariate analysis of variation of the effects of pollinators, floral manipulation treatments and years on pollinator visitation rate and duration.

Parameters	Sources	MS	d.f.	*F*	*P*
Visitation rate	Pollinators	5.508	4	7.336	0.007**
	Treatments	2.583	1	3.440	0.097
	Years	2.450E-6	1	0.000	0.999
	Pollinators × Treatments	1.214	4	0.404	0.801
	Pollinators × Years	1.105	4	2.798	0.092
	Treatments × Years	0.126	1	0.069	0.796
Visitation duration	Pollinators	250.748	4	95.913	0.000***
	Treatments	20.120	1	7.696	0.022*
	Years	4.104	1	1.570	0.242
	Pollinators × Treatments	5.638	4	0.539	0.711
	Pollinators × Years	4.996	4	4.897	0.023*
	Treatments × Years	1.142	1	0.046	0.834

d.f., degrees of freedom; MS, mean squares. *P<0.05, **P<0.01, ***P<0.001.

**Figure 4:**
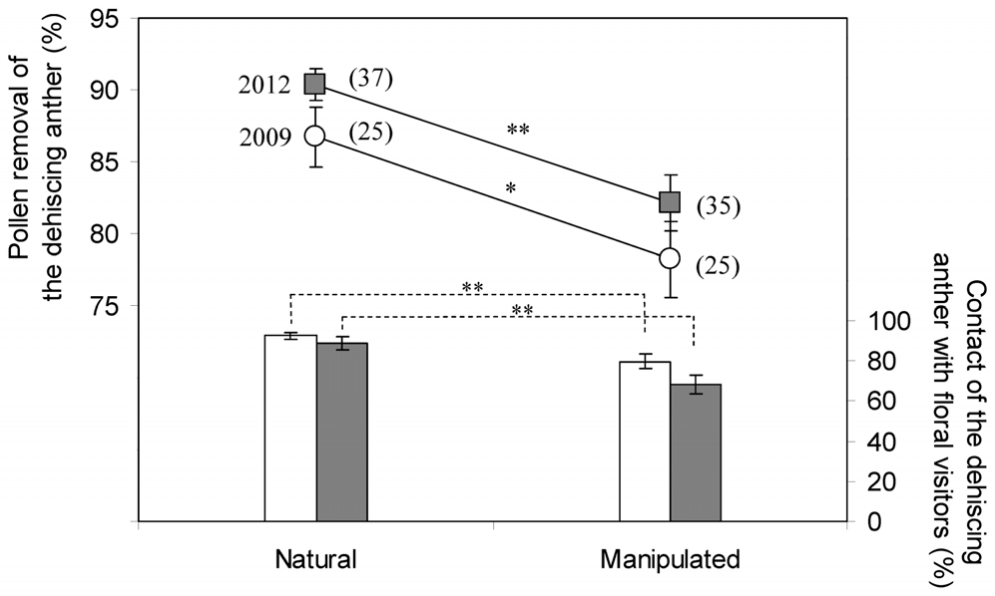
Pollination consequences of stamen movement manipulations in two different years. Data are Mean ± SE (“*” P<0.05; “**” P<0.001. Student's t-test for pollen removal and one-way ANOVA for contact frequency). Numbers in brackets indicate sample size.

Control flowers (i.e. flowers with free movement of stamens) were visited by different groups of pollinators with high visitation rates and duration (Fig. 3). As a result, most pollen grains of the second-moved stamen were successfully shed, with an average value of 30 911±3 666 (more than 85% of the pollen grains in the anther) removed in 2009 (N = 25) and 56 609±4 149 (>90%) in 2012 (N = 37; three of the 40 control flowers were lost; Fig. 4).

When the first stamen was fixed at the flower's center after its free filament elongation and anther dehiscence, the number of insect visits per hour (visitation rate) was not affected (one-way ANOVA, *P*>0.025; Fig. 3, Table 2). But, the visitation durations of hoverflies and vespid wasps decreased dramatically (one-way ANOVA, *P*<0.01 and *P*<0.005 for hoverflies in 2009 and 2012, respectively; *P*<0.025 and *P*<0.01 for vespid wasps in 2009 and 2012, respectively; Fig. 3) and the visitation duration of ichneumon wasps decreased significantly in 2009 (one-way ANOVA, *P*<0.025. Fig. 3). When the second stamen elongated its filament, its dehiscing anther was located under or very close to the first dehisced anther (Fig. S2). Consequently, floral visitors contacted the dehiscing anther less often (Fig. 4), and only 27 877±4 720 (78.2%) and 51 442±7 187 (82.1%) of the pollen grains were removed from this anther in 2009 and 2012, respectively. Compared with the control (natural condition), the percentage of pollen removed showed a significant decrease (Student's *t*-test; *P*<0.025, N = 25 and *P*<0.001, N = 35 in 2009 and 2012, respectively. Five of the 40 manipulated flowers were lost in 2012; Fig. 4).

## Discussion

This study suggests that one-by-one stamen movement in *Parnassia palustris* reflects an adaptation to decrease interference between dehisced and dehiscing anthers, which probably could promote pollen exports. The results demonstrate that the evolution to decrease anther-anther interference should be recognized as an important selective force in floral evolutions.

### Patterns of one-by-one stamen movement

One-by-one stamen movement in *P. palustris* is characterized by two striking phenomena: (i) The five fertile stamens elongate their filaments one-by-one and anthers dehisce sequentially at the floral center; (ii) the anther-dehisced stamen will bend out of the floral center before the next stamen's anther dehiscence. The stamens move very slowly, with filament elongation taking nearly a whole day to reach their maximum. Anther dehiscence lasts for approximately 5 h and finally, the filament deflexion lasts for another day (Fig. 1, Table 1). These movements are obviously much slower than in other taxa. In Loasaceae the stamens move from their original places (petals) to the floral center in only 2–4 min [5], [20] and in Rutaceae this movement takes less than 20 min [18]. The higher frequency of pollinator visits [5], [18] and stamen movements can be triggered by floral visitors in Loasaceae [5], [20], [21] are probably the main reasons for the quicker movement of stamens in these taxa. In *P. palustris* however, the open habitats with densely coexisting plants make pollinator visits highly variable (Fig. 3) and the longer presentation of pollen (anther dehiscence) and slow movement of stamens are probably selected for to enhance pollen exports.

### Pollination adaptations of one-by-one stamen movement

Individual dehiscence of anthers in *P. palustris* provides pollen in small doses to the pollinators. This could be regarded as one form of pollen ‘packaging’ and ‘dispensing’ mechanisms according to ‘pollen presentation theory’ [6], [7], [8], [9], [10]. When the dehisced anther is manipulated to remain at the floral center, the pollen removal of the next dehiscing anther decreases significantly (Fig. 4). There are at least two explanations for this result: (1) the co-presence of the dehisced anther at the flower center decreases the contact frequency of pollinators with the dehiscing anther (Fig. 4); (2) the dehisced anther has little fresh pollen in it and may discourage the ‘hungry’ pollen-collecting insects such as hoverflies and wasps, resulting in decreased visitation durations (Fig. 3) and suppressed pollen dispersal of the dehiscing anther (Fig. 4). We therefore conclude that deflection of the anther-dehisced stamen from the flower's center in *P. palustris* flowers is likely a mechanism to avoid interference with late-dehiscing anthers. The alternate movement sequence of the five fertile stamens (Fig. 1E) and the abscission of the dehisced anther after moving out of the floral center (Fig. 1D,E) may further decrease anther-anther interferences. During this process, the stigma is getting mature and selfing is possible since this plant is self-compatible (Fig. 2). We speculated that the drop off of the dehisced anthers may also avoid anther-stigma interferences (including selfing) and thus may be selectively advantageous. A detailed study is needed to confirm this hypothesis.

At present, one-by-one stamen movement is reported in four species-rich families, Loasaceae [5], [19], Celastraceae [16], Tropaeolaceae [17], and Rutaceae [18]. In Celastraceae, not only *Parnassia* but also several other genera such as *Brexia* and *Hippocratea* show sequential movement of the stamens during anthesis [16]. Ren [17] found that most of these flowers are radially symmetrical, protandrous (male organs mature before female organs) and generalist-pollinated. In these less-specialized flowers, the pollen placements on the floral visitors is not precise and can cause considerable pollen loss, while reducing interferences among anthers and stigmas may be a way to compensate for that [11], [12], [13], [14]. Thus protandry is probably selected for to reduce anther-stigma interferences [11], [13], [14], while one-by-one stamen movement (anther dehiscence) could decrease the anther-anther interference and pollen wastages.

### Other mechanisms for decrease in anther-anther interference

Selection to decrease anther-anther interference possibly is also involved in the evolution of some floral traits traditionally explained as pollen packaging or dispensing mechanism. For example, the separation of fertile anthers into different heights within the same flower is not rare in angiosperms, such as didynamous and tetradynamous stamens [26], [27] and anther height dimorphism in tristylous species [28]. Spatial separation of anthers is often associated with temporally-separated dehiscence [27], [28] and interaction with different pollinators [27], [28], [29]. In fact, the long pair of anthers in the didynamous stamens normally dehisces first and then moves apart to the sides of the floral tube [30], [31], suggesting an adaptation to reduce interferences with pollen dispersals of the short pair. In the didynamous stamens of the bumblebee-pollinated *Incarvillea arguta* (Bignoniaceae), Han et al. [32] reported an interesting floral trait that anther appendages of one set of anthers can be triggered to release pollen by the direction the bumblebee moves into the flower, while the other set is only elicited by the opposite direction (exiting the flower) [32]. Such differentiation in pollen release cannot decrease single-visit pollen removal and thus it is not an effective pollen dispensing mechanism. However, it seems to be effective in reducing the interference of pollen dispersal between the two sets of anthers.

The adaptation of anther separation in tetradynamous stamens is not very clear [33]. Kudo [26] found in *Brassica rapa* that, when the four long stamens were manipulated to be two, pollen dispersals increased significantly. This result suggests higher anther-anther interference in tetradynamous stamens than in didynamous stamens and is perhaps one of the evolutionary causes for the restricted occurrence of tetradynamous stamens in Brassicaceae, which further suggests that reducing anther-anther interference may play a role in shaping floral structures.

Taken together, our data reveal that one-by-one stamen movement in the generalist-pollinated *Parnassia palustris* may promote pollen removal by presenting pollen gradually to pollinators and, more importantly, by decreasing interferences between dehisced and dehiscing anthers. We thus provide direct experimental evidences for such ‘anther-anther interference’ in angiosperms and its role in driving the evolution of floral traits traditionally explained as pollen dispensing mechanisms. The great majority of angiosperms have flowers with many stamens, so there is far more scope for the operations of anther-anther interferences. Future studies should recognize these kinds of selective forces, which might interact with anther-stigma interferences, when interpreting floral adaptations and evolutions.

## Supporting Information

Figure S1 **Main floral visitors of** ***Parnassia palustris*** **in the northeast China.** Five functional groups are identified: hoverflies (Syrphidae spp., including A, B, C, and D); Ichneumon wasps of *Ichneumonidae* sp. (E); Vespid wasps of *Vespidae* sp. (F); Ants of *Camponotus* sp. (G); Flies including *Muscidae* sp. (H) and *Calliphoridae* sp. (I).
(TIF)

Figure S2 **Experimental manipulation of stamen movement in** ***Parnassia palustris*** **.** Manipulation is made by binding the filament of the first-moved stamen with a white thread to the pedicel to fix it at the floral center after free filament elongation and anther dehiscence (A, top view; B, side view; C, a manipulated flower). The control flower is also tethered with a white thread at the pedicel (D) to minimize possible effects of floral manipulation.
(TIF)

Table S1 **Pollen and ovule production of** ***Parnassia palustris*** **flower in 2009 and 2012.** Data are means with Mean ± S.E.. Different letters for the same column indicate significant difference (Student's t-test, *P*<0.001).
(DOC)
