# Supplementary figures and images for: Is There ‘Anther-Anther Interference’ within a Flower? Evidences from One-by-One Stamen Movement in an Insect-Pollinated Plant
Source: PLoS One. 2014 Jan 27;9(1):e86581. doi: 10.1371/journal.pone.0086581 (PMC3903572; doi:10.1371/journal.pone.0086581)

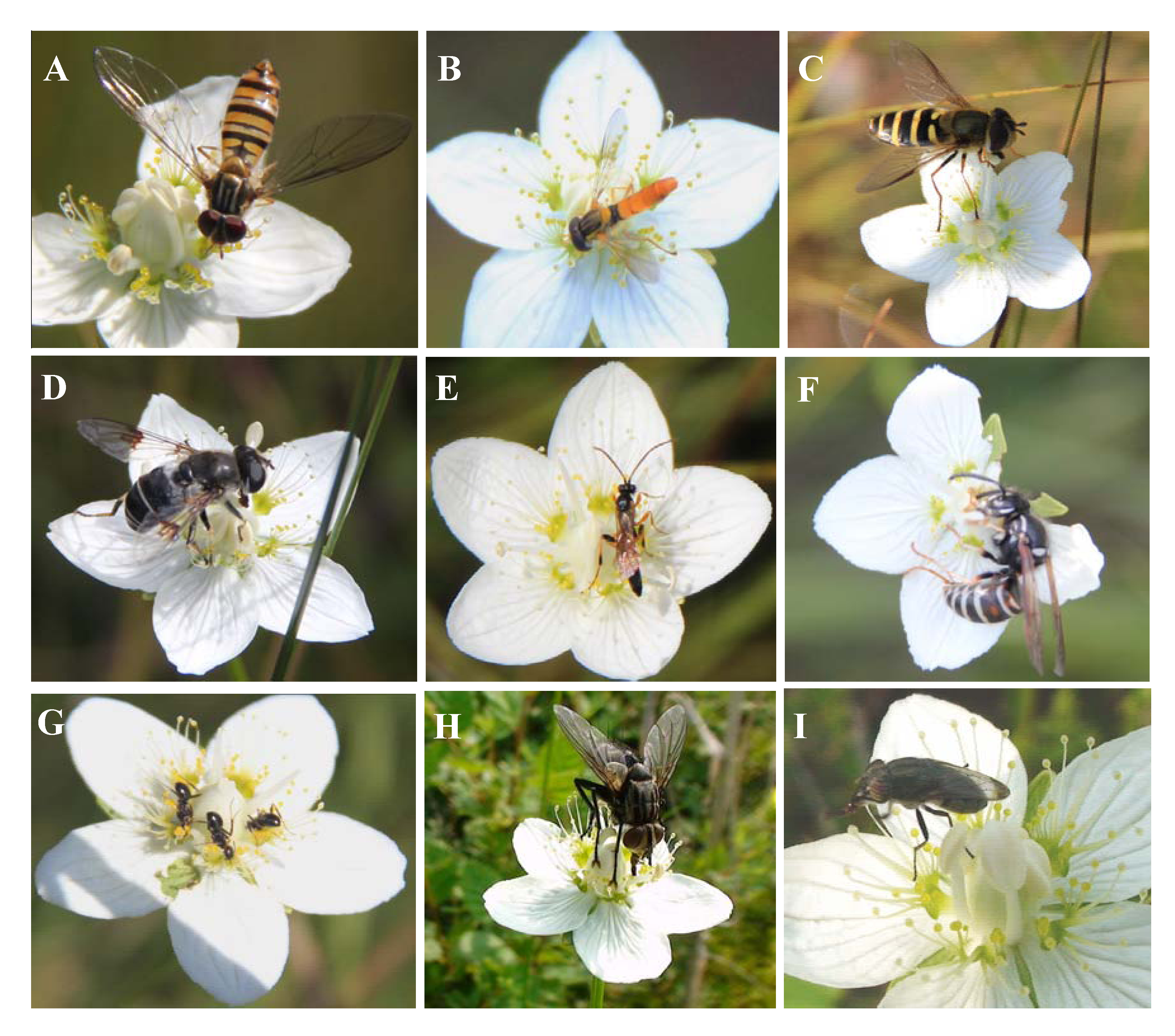

Supplement: Figure S1 — Main floral visitors of Parnassia palustris in the northeast China. Five functional groups are identified: hoverflies (Syrphidae spp., including A, B, C, and D); Ichneumon wasps of Ichneumonidae sp. (E); Vespid wasps of Vespidae sp. (F); Ants of Camponotus sp. (G); Flies including Muscidae sp. (H) and Calliphoridae sp. (I). (TIF) [file pone.0086581.s001.tif]

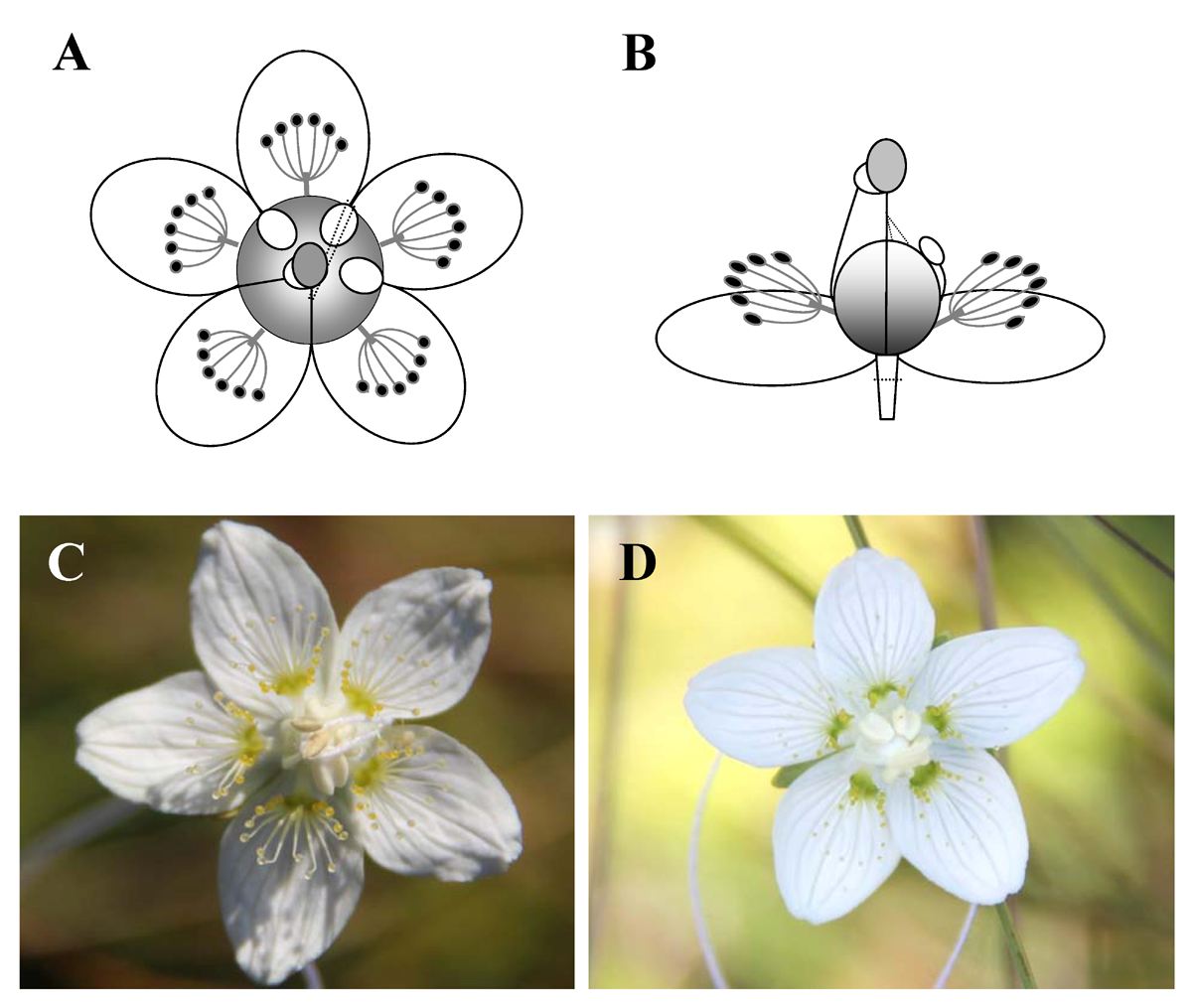

Supplement: Figure S2 — Experimental manipulation of stamen movement in Parnassia palustris . Manipulation is made by binding the filament of the first-moved stamen with a white thread to the pedicel to fix it at the floral center after free filament elongation and anther dehiscence (A, top view; B, side view; C, a manipulated flower). The control flower is also tethered with a white thread at the pedicel (D) to minimize possible effects of floral manipulation. (TIF) [file pone.0086581.s002.tif]
