# Supplementary material for: Is There ‘Anther-Anther Interference’ within a Flower? Evidences from One-by-One Stamen Movement in an Insect-Pollinated Plant
Source: PLoS One. 2014 Jan 27;9(1):e86581. doi: 10.1371/journal.pone.0086581 (PMC3903572; doi:10.1371/journal.pone.0086581)
Supplement: Table S1 — Pollen and ovule production of Parnassia palustris flower in 2009 and 2012. Data are means with Mean ± S.E.. Different letters for the same column indicate significant difference (Student's t-test, P<0.001). (DOC) [file pone.0086581.s003.doc]

| **Table S1. Pollen and ovule production of *Parnassia palustris* flower in 2009 and 2012.** Data are Mean ± S.E.. Different letters for the same column indicate significant difference (Student’s *t*-test, *P* < 0.001) | | | | | | | | |
| --- | --- | --- | --- | --- | --- | --- | --- | --- |
| Year | Pollen number for each anther | | | | | Total pollen number of the five anthers | Ovule number | Pollen : Ovule Ratio |
|  | 1st-moved | 2-nd moved | 3-rd moved | 4-th moved | 5-th moved |  |  |  |
| 2009 | 50253 ± 2645 a | 49464 ± 2249 a | 47958 ± 2760 a | 48403 ± 2523 a | 47890 ± 2467a | 243969 ± 11415 a | 257 ± 10 a | 978 ± 60 a |
| 2012 | 59166 ± 4943 a | 65123 ± 3228 b | 69405 ± 4313 b | 52835± 4914 a | 66645 ± 6187 b | 313175 ± 13189 b | 335 ± 41 a | 1189 ± 165 a |
